# Supplementary material for: The Role of Airborne Particles in the Epidemiology of Clade 2.3.4.4b H5N1 High Pathogenicity Avian Influenza Virus in Commercial Poultry Production Units
Source: Viruses. 2023 Apr 19;15(4):1002. doi: 10.3390/v15041002 (PMC10142477; doi:10.3390/v15041002)
Supplement: Supplementary file 1 [file viruses-15-01002-s001.zip › viruses-2327765-supplementary.pdf]

Supplementary Tables

Table S1. IP1 ducks: H5 HPAIV RNA detection and virus isolation from air and environmental samples collected on and around the duck IP

**Sample number**	**Sample type**	**House**	**Location**	**H5 HPAIV RRT-PCR Ct value***	**Volume of air collected (liters)**	H5 HPAIV RNA Detected	**Virus isolated**
1	Air	1	Inside house 1 (during culling)	30.88	104	Yes	Yes
2	Air	2	Inside house 2 (normal operation)	29.57	120	Yes	Yes
3	Air	1	North-East outside house 1 (within 1 m)	29.06	471	Yes	Yes
4	Air	1	North outside house 1 (within 1 m)	33.00	330	Yes	No
5	Air	2	North-East outside house 2 (within 1 m)	34.45	590	Yes	No
6	Air	2	North outside house 2 (within 1 m)	No Ct	522	No	No
7	Air	N/A	10m North-East (downwind)	32.67	515	Yes	No
8	Air	N/A	120m North North-East (downwind)	No Ct	494	No	No
9	Vent dust	1	North-West side of house 1	28.27	N/A	Yes	No
10	Vent dust	1	North-East side of house 1	30.05	N/A	Yes	ND
11	Vent dust	1	East side of house 1	32.48	N/A	Yes	ND
12	Vent dust	1	South-East side of house 1	30.57	N/A	Yes	No
13	Vent dust	1	South-West side of house 1	31.42	N/A	Yes	ND
14	Vent dust	1	West side of house 1	31.40	N/A	Yes	No
15	Vent dust	2	North-West side of house 2	31.15	N/A	Yes	No
16	Vent dust	2	North-East side of house 2	32.53	N/A	Yes	ND
17	Vent dust	2	East side of house 2	33.53	N/A	Yes	ND
18	Vent dust	2	South-East side of house 2	26.39	N/A	Yes	Yes
19	Vent dust	2	South-West side of house 2	27.18	N/A	Yes	No
20	Vent dust	2	West side of house 2	No Ct	N/A	No	ND
21	Ground water	1	South entrance to house 1	No Ct	N/A	No	ND
22	Ground water	2	South entrance to house 2	No Ct	N/A	No	ND
23	Duck feathers	2	East outside house 2	33.59	N/A	Yes	No
24	Wet straw	2	North-East side of house 2	No Ct	N/A	No	ND
25	Wet straw	2	East side of house 2	No Ct	N/A	No	ND
26	OP swab	2	Inside house 2	13.23	N/A	Yes	Yes

* Ct values derived from the H5 HPAIV RRT-PCR as previously described (72); samples with Ct values ≥36 were considered negative; lower Ct values indicate higher levels of H5 HPAIV RNA; N/A = not applicable; ND = virus isolation not performed.

Page Break

Table S2. IP2 turkeys: H5 HPAIV RNA detection and virus isolation from air and environmental samples collected on and around the IP

Sample number	Sample Type	house	Location	H5 HPAIV RRT-PCR Ct value*	Volume of air collected (liters)	H5 HPAIV RNA detected	Virus isolated
1	Air	2	Inside	32.91	338	Yes	No
2	Air	3	Inside	33.11	372	Yes	Yes
3	Air	1	Outside North-East within 1m	No Cq	484	No	ND
4	Air	3	Outside North-East within 1m	No Cq	461	No	ND
5	Air	3	Outside East-Northeast under extract	35.55	431	Yes	No
6	Air	N/A	50m North-East of houses	No Cq	568	No	ND
7	Air	N/A	50m North-East of carcass loading area	No Cq	542	No	ND
8	Air	N/A	120m North-East of houses	No Cq	588	No	ND
9	Feather	N/A	60m North-East of carcass loading area	35.11	N/A	Yes	No
10	Feather	N/A	65m North-East of carcass loading area	30.63	N/A	Yes	No
11	Feather	N/A	60m North-North-East of carcass loading area	No Cq	N/A	No	ND
12	Feather	N/A	65m North-North-East of carcass loading area	No Cq	N/A	No	ND
13	Feather	N/A	80m North-East of carcass loading area	30.06	N/A	Yes	No
14	Feather	N/A	100m North-East of carcass loading area	No Cq	N/A	No	ND
15	Feather	N/A	120m North-East of carcass loading area	No Cq	N/A	No	ND
16	Feather	N/A	150m North-East of carcass loading area	No Cq	N/A	No	ND
17	Feather	N/A	160m North-East of carcass loading area	No Cq	N/A	No	ND
18	Feather	N/A	180m North-East of carcass loading area	No Cq	N/A	No	ND

19	Feather	N/A	180m North-East of carcass loading area	No Cq	N/A	No	ND
20	Feather	N/A	180m North-East of carcass loading area	No Cq	N/A	No	ND
21	Dust	1	Inside	33.39	N/A	Yes	No
22	Dust	1	Outside East	36.54	N/A	No	ND
23	Dust	1	outside North-East	35.75	N/A	Yes	No
24	Dust	1	Inside	34.76	N/A	Yes	ND
25	Dust	2	Outside East	34.81	N/A	Yes	No
26	Dust	2	Outside East-Northeast	No Cq	N/A	No	ND
27	Dust	2	Inside North-West	27.96	N/A	Yes	No
28	Dust	2	Inside	36.62	N/A	No	ND
29	Dust	2	Outside South-East	36.07	N/A	No	ND
30	Dust	3	Outside East	No Cq	N/A	No	ND
31	Dust	3	Outside East-Northeast	33.28	N/A	Yes	No
32	Dust	3	Inside extract North-West	28.72	N/A	Yes	No
33	Dust	3	Inside	34.2	N/A	Yes	No
34	Dust	3	Outside South-East	34.7	N/A	Yes	ND
35	Water	1	Outside East	No Cq	N/A	No	ND
36	Water	3	Outside South-Southeast	38.72	N/A	No	ND
37	Water	3	East-Northeast	37.21	N/A	No	ND
38	Water	N/A	Middle of IP	38.92	N/A	No	ND
39	Water	3	Inside	31.68	N/A	Yes	ND
40	Water	3	Outside East	34.91	N/A	Yes	No
41	Op swab	2	Inside	26.85	N/A	Yes	Yes
42	Op swab	3	Inside	25.27	N/A	Yes	Yes

* Ct values derived from the H5 HPAIV RRT-PCR as previously described (48); samples with Ct values ≥36 were considered negative; lower Ct values indicate higher levels of H5 HPAIV RNA; N/A = not applicable; ND = virus isolation not performed.

Page Break

Table S3. IP3 chickens: H5 HPAIV RNA detection and virus isolation from air and environmental samples collected on and around the IP

Sample number	Sample Type	House	Location	H5 HPAIV RRT-PCR Ct value*	Volume of air collected (liters)	Interpretation	Virus isolated
1	Air	1	Inside	38.07	195	No	No
2	Air	5	Inside	35.46	187	Yes	No

3	Air	1	Outside North-East roof	No Cq	323	No	No
4	Air	5	Outside North-East roof	37.24	192	No	No
5	Air	5	Outside North-East vent	38.11	165	No	No
6	Air	5	Outside North-East within 5m	38.34	568	No	No
7	Air	5	Outside North-East within 25m	No Cq	830	No	No
8	Air	5	Outside North-East within 70m	No Cq	765	No	No
9	Feather	1	Inside	No Cq	N/A	No	ND
10	Feather	5	Inside	31.35	N/A	Yes	No
11	Feather	1	Outside 10m North-East	No Cq	N/A	No	ND
12	Feather	5	Outside 10m West	28.86	N/A	Yes	No
13	Feather	5	Outside 5m West	39.42	N/A	No	No
14	Feather	5	Outside 50m North-East	39.7	N/A	No	ND
15	Feather	5	Outside 60m North-East	No Cq	N/A	No	ND
16	Dust	1	Outside vent South-East	No Cq	N/A	No	ND
17	Dust	1	Outside vent South	No Cq	N/A	No	ND
18	Dust	1	Outside vent South-West	38.67	N/A	No	ND
19	Dust	1	Outside vent West	No Cq	N/A	No	ND
20	Dust	1	Outside vent West	No Cq	N/A	No	ND
21	Dust	1	Outside vent North-West	No Cq	N/A	No	ND
22	Dust	1	Outside vent North	No Cq	N/A	No	ND
23	Dust	1	Outside vent North-East	No Cq	N/A	No	ND
24	Dust	1	Outside vent East	No Cq	N/A	No	ND
25	Dust	5	Outside vent South-East	No Cq	N/A	No	ND
26	Dust	5	Outside vent South	No Cq	N/A	No	ND

27	Dust	5	Outside vent South-West	32.61	N/A	Yes	No
28	Dust	5	Outside vent West	No Cq	N/A	No	ND
29	Dust	5	Outside vent West	No Cq	N/A	No	ND
30	Dust	5	Outside vent West	No Cq	N/A	No	ND
31	Dust	5	Outside vent North-West	No Cq	N/A	No	ND
32	Dust	5	Outside vent North	37.38	N/A	No	No
33	Dust	5	Outside vent North-East	No Cq	N/A	No	ND
34	Dust	1	Inside vent South-East	No Cq	N/A	No	ND
35	Dust	1	Inside vent South	No Cq	N/A	No	ND
36	Dust	1	Inside vent South-West	No Cq	N/A	No	ND
37	Dust	1	Inside vent Centre	39.86	N/A	No	ND
38	Dust	5	Inside vent South-East	No Cq	N/A	No	ND
39	Dust	5	Inside vent South	39.08	N/A	No	ND
40	Dust	5	Inside vent South	No Cq	N/A	No	ND
41	Dust	5	Inside vent East	No Cq	N/A	No	ND
42	Dust	5	Inside vent Centre	No Cq	N/A	No	ND
43	Water	1	Inside	No Cq	N/A	No	ND
44	Water	5	Inside	34.14	N/A	Yes	No
45	Bird faeces	5	Outside 50m North-East	No Cq	N/A	No	ND
46	Op swab	1	Inside	No Cq	N/A	No	ND
47	Cl swab	1	Inside	No Cq	N/A	No	ND
48	Op swab	5	Inside	24.48	N/A	Yes	Yes
49	Cl swab	5	Inside	28.06	N/A	Yes	ND

* Ct values derived from the H5 HPAIV RRT-PCR as previously described (49); samples with Ct values ≥36 were considered negative; lower Ct values indicate higher levels of H5 HPAIV RNA; N/A = not applicable; ND = virus isolation not performed.
